# Relationships of emerging biomarkers of cancer cachexia with quality of life, appetite, and cachexia

**DOI:** 10.1007/s00520-024-08549-5

**Published:** 2024-05-14

**Authors:** M. Lipshitz, J. Visser, R. Anderson, DG. Nel, T. Smit, HC. Steel, BL. Rapoport

**Affiliations:** 1 Division of Human Nutrition, Stellenbosch University, Stellenbosch, 7600 South Africa; 2 Melanie Levy Dietician, Johannesburg, 2192 South Africa; 3 Department of Immunology, School of Medicine, Faculty of Faculty of Health Sciences, University of Pretoria, Pretoria, 001 South Africa; 4 Centre for Statistical Consultation, Stellenbosch University, Stellenbosch, South Africa; 5 The Medical Oncology Centre of Rosebank, Johannesburg, South Africa

**Keywords:** Anorexia, Appetite, Biomarkers, Cachexia, Quality of life

## Abstract

**Purpose:**

Quality of life (QoL), appetite, cachexia, and biomarkers [albumin, hemoglobin (Hb), neutrophils, lymphocytes, platelets, C-reactive protein (CRP), tumor necrosis factor alpha (TNFα), interleukin 6 (IL-6), interleukin 8 (IL-8), C-X-C motif chemokine ligand 5 (CXCL5) and citrullinated histoneH3 (H3Cit)] were compared for 40 cases with advanced cancer and 40 healthy controls. Baseline differences and significant relationships were explored for biomarkers with QoL, appetite, and cachexia.

**Methods:**

In a prospective case–control, age and sex matched study, the European Organisation for the Research and Treatment of Cancer Quality of Life–C30 questionnaire (EORTC-QLQ-C30) for QoL, the Functional Assessment of Anorexia and Cachexia Therapy assessment (FAACT A/CS-12) for appetite, and a five-factor cachexia assessment tool for cachexia assessment were performed. Routine hematological measurements and blood chemistry analyses together with ELISA procedures and a Multiplex® bead array platform, were used for biomarker analysis. Descriptive statistics and regression analyses were undertaken. *P* < 0.05 defined statistical significance.

**Results:**

Global health status (QL-G), functional scales (QL-FS), and symptom scales (QL-SS) differed for cases and controls (*p* < 0.01). In cases, differences were observed for QL-G (*p* < 0.01), QL-FS (*p* < 0.01), and QL-SS (*p* = 0.01) compared to standardized references values. FAACT A/CS-12 scores differed significantly between cases and controls (*p* < 0.01) and 30% of cases scored “poor” appetites. Cachexia was present in 60% of cases. Albumin, lymphocytes, platelets, Hb, platelet to lymphocyte ratio (PLR), systemic immune-inflammation index (SII), CRP, TNFα, all at *p* < 0.01, neutrophil to lymphocyte ratio (NLR) (*p* = 0.02), IL-6 (*p* < 0.04), and IL-8 (*p* = 0.02) differed significantly between cases and controls. No difference was found for CXCL5 or H3Cit. Albumin NLR, Hb, PLR, SII, TNFα, IL-8, and CRP showed significant relationships with all aspects of QoL. QL-FS was significantly related to CXCL5 (*p* = 0.04), significant relationships with FAACT A/CS-12 included: NLR (*p* = 0.002), Hb (*p* < 0.001), and PLR (*p* < 0.01). NLR, PLR, SII, TNFα, IL-6, IL-8, and CRP correlated positively to cachexia and albumin while Hb and lymphocyte count correlated negatively to cachexia.

**Conclusion:**

CXCL5 and H3Cit were not reliable biomarkers for cancer cachexia, nor significantly related to QoL, appetite or cachexia. Albumin, NLR, Hb, PLR, SII, TNFα, IL-8, and CRP were reliable indicators of QoL, appetite, and cachexia. Future research should include other novel biomarkers namely growth differentiation factor-15 (GDF-15), fibroblast growth factor 21 (FGF-21), fractakline, interferon gamma (IFN-y), IL-16, macrophage colony stimulating factor (M-CSF), and macrophage procoagulant–inducing factor (MPIF).

**Supplementary Information:**

The online version contains supplementary material available at 10.1007/s00520-024-08549-5.

## Background and introduction

Cancer cachexia is defined as a multi-factorial and complex metabolic syndrome [1] characterized by an ongoing loss of skeletal muscle mass that cannot be fully reversed by conventional nutritional support and leads to progressive functional impairment [2]. The role of proper cachexia staging and identifying the physiological signs of progressing cachexia, ensures a goal-directed and individualized approach that allows for early nutritional, metabolic or pharmacological treatment [3].

Anorexia assessment is instrumental in identifying the early diagnosis of pre-cachexia and cachexia, where cachexia is still responsive to treatment and nutrition interventions [4]. Weight loss and a compromised nutritional status have profound negative effects on the QoL of cancer patients [5], supporting the need for timeous interventions of cachexia management and early diagnoses of anorexia, reduced QoL, and cachexia presence.

In advanced cancer, anorexia is the fourth most common symptom after pain, fatigue, and weakness [6], with a prevalence of 40% at diagnosis and 70% in advanced disease [7]. Appetite loss and fatigue may not only compromise nutritional status but may also decrease health status and QoL and ultimately result in unplanned interruptions of chemotherapy drug administration, with subsequent increases in morbidity and mortality [1]. Additionally, unplanned weight loss results in poor patient psychosocial well-being which impacts on self-esteem, with cachexia/malnutrition being a leading cause of hospitalization [8].

Validated questionnaires are pivotal for anorexia diagnosis, linking anorexia to cachexia status and ensuring targeted treatment [9]. Cachexia assessments incorporating elements of weight loss, inflammation, appetite, sarcopenia, and performance status are preferable as opposed to the use of BMI or percentage weight loss alone [4, 10]. Quality of life analysis, encompasses psychological well-being, functional status, health perceptions, and disease and treatment-related symptoms, which through the availability of validated questionnaires has been utilized in multicultural research settings [11], in which its categorical assessments have been associated with survival and skeletal muscle mass [12]. The combination of anorexia, QoL, and comprehensive cachexia assessment tools is invaluable in directing clinicians to optimize and identify the early management of cancer cachexia.

Currently, there are no reliable laboratory tests that categorically link biomarkers to the diagnosis of cachexia. Biomarkers found to be elevated in cancer cachexia underpin the metabolic derangements that drive the cachexia process and in turn may result in both a compromised appetite and QoL [13]. Hypothalamic exposure to inflammatory cytokines (often secreted from tumors) results in systemic inflammation and consequently anorexia, weight loss and skeletal muscle atrophy. Additionally, these cytokines result in changing central nervous system (CNS) outputs with the release of adrenal corticosteroids ultimately increasing anorexia and fatigue [14].

Targeting these biomarkers and an improved understanding of their roles may assist in the improvement of cachexia management. Interleukin 6 for example, directly impacts leptin and ghrelin, which in turn contributes to the depressed appetite in cancer cachexia patients. Ghrelin agonists alone may be an effective target to combat cancer cachexia. Additionally, elevated levels of growth differentiation factor 15 (GDF15) in cancer cachexia patients may be responsible for anorexia as recombinant GDF15 reduces food intake and promotes weight loss via anorexia [13].

These biomarkers show promise in current ongoing clinical trials designed to determine which biomarker or combination of biomarkers may be best suited to target cachexia and mitigate the resultant anorexia and QoL declines experienced in cancer patients. Predictive biomarkers to detect pre-cachexia (prior to appetite and skeletal muscle loss), prognostic biomarkers (indicating those that may respond to cachexia treatment) and reliable biomarkers to stage cachexia severity have yet to be validated [8], necessitating the need for further research, particularly of the less-well understood biomarkers.

In this context, there is paucity of data with respect to the roles and relationships that emerging biomarkers play in cancer cachexia. For example, C-X-C motif chemokine ligand 5 (CXCL5), has been linked to inflammation [15], metastasis [16], and cancer progression and has been researched as a potential biomarker for therapeutic targeting [17]. Additionally, research has shown a relationship between the systemic inflammation of cancer, neutrophil activation, and the release of citrullinated histone H3 (H3Cit). Indeed, levels of H3Cit in cancer patients have been shown to have a positive association with TNFα, IL-6, and IL-8, indicating a relationship between the expression of these pro-inflammatory cytokines and the release of H3Cit [18]. H3Cit may therefore also be considered as a potentially valuable biomarker to be targeted to mitigate the inflammation, cachexia and the sequelae thereof.

Associations of several cytokines with anorexia, QoL, and cachexia have been established. However, regarding emerging biomarkers of cancer cachexia, such relationships are largely unknown, and the ideal biomarkers have yet to be determined, thus warranting further research between emerging biomarkers of cancer cachexia with validated QoL, anorexia, and cachexia assessments.

## Methods

### Study population

Forty patients with advanced stage 4 cancer of various types, and 40 healthy age and gender-matched controls identified, using purposive sampling, were studied in a prospective case control design. Patients were recruited in the setting of a private oncology practice. Participants were recruited from March 2020 until March 2021.

Adults older than 18 years, presenting with a diagnosis of stage 4 cancer of different types were deemed as being eligible cases. For all participants, confounding conditions that excluded eligibility were severe or chronic illnesses of the liver, chronic kidney disease, inflammatory gastrointestinal tract disorders (ulcerative colitis, Crohn’s disease), chronic obstructive pulmonary disease, congestive heart failure, insulin-dependent diabetes mellitus, active uncontrolled infection, neuromuscular disorders with hemiplegia, rheumatoid arthritis affecting the hands, and patients not consenting to take part in the study. Controls were healthy individuals—self-reported to be healthy and not taking any chronic medications pertaining to the relevant exclusions.

### Biomarker collection, processing, and storage

Ten milliliters of blood were drawn per blood collection tube from each participant, where one ethylene diamine tetra acetic acid (EDTA)–containing tube was used for full blood count analysis, one serum separator tube (SST) was used for serum albumin determinations, and one EDTA containing tube was processed for plasma used for the investigational markers.

Biomarker analyses were performed on the same cohort of cancer patients and healthy control individuals as reported previously [19]. Biomarkers investigated included serum albumin, Hb, neutrophils, lymphocytes, platelets and the investigational markers C-reactive protein (CRP), interleukin (IL)-6, IL-8, TNFα, H3Cit, and CXCL5. Neutrophil to lymphocyte ratio (NLR), platelet to lymphocyte ratio (PLR) ratio, and the systemic immune inflammation index [platelets (× 10^9^/L) × NLR] (SII) were included in inflammation assessments.

The full blood count (FBC) and serum albumin vacutainers were analyzed immediately on the day of collection by an on-site commercial laboratory (Lancet Laboratories©). Plasma samples were prepared within 30 min of venepuncture by centrifuging the EDTA-containing blood collection tubes at 1500 × *g* for 10 min (Kendro Laboratory Products GmbH, Postfach 15 63, D-63405 Hanau). Following processing, plasma samples for investigational markers were stored at − 80 °C until use.

Reference ranges for routine makers were standardized, accepted reference ranges used by commercial laboratories. Cut-offs used for NLR, PLR, SII, and investigational markers were defined according to the cut-offs yielded from receiver operating characteristic (ROC) curve analysis.

Briefly, TNFα, IL-6, and IL-8 concentrations were measured using a MILLIPLEX Map Cytokine/Chemokine kit (Merck KGaA, Darmstadt, Germany). Circulating levels of CXCL5 levels were determined using an InVitrogen ProcartaPlex® multiplex assay (Thermo Fisher Scientific, Inc.). The assays were conducted according to the protocol supplied by the manufacturers and a Bio-Plex suspension bead array platform (Bio-Rad Laboratories Inc., Hercules, CA, USA) together with Bio-Plex Manager software (Version 6.0) was used for bead acquisition and analysis of median fluorescence intensity. The results are reported as picograms (pg)/mL.

The Clone11D3 enzyme-linked immunosorbent assay (ELISA) kit (Cayman Chemical Co., Ann Arbor, MI, USA) was used to measure levels of H3Cit [20]. Following the processing of the ELISA as outlined by the manufacturer, the optical density for each sample was measured spectrophotometrically at 450 nm (BioTek Inc., Winooski, VT, USA), and the final concentration of each sample was determined from the generated curve using GraphPad Prism 5 (GraphPad Software, Inc, San Diego, CA, USA). The results are presented as nanograms (ng)/mL.

The CardioPhase hsCRP test kit (Siemens Healthcare Diagnostics, Midrand, Johannesburg, South Africa) was employed to determine plasma CRP concentrations using the Attelica 630N nephelometer (Siemens, MU, Germany). The CRP concentrations are reported as milligrams (mg)/L.

### Anthropometric assessment

Anthropometric assessments included weight and height measurement, body mass index (BMI) calculation, and self-reported weight loss of the cases which was recorded.

### Appetite assessment

The Functional Assessment of Anorexia and Cachexia Therapy assessment (FAACT A/CS-12) Version 4 assessment was self-administered for all participants. The questionnaire consisting of 12 questions was scored on a five-point Likert scale [ranging from 0 (not at all) to 4 (very much)]. The FAACT A/CS-12 was summated using the standard prescribed method [9]. The total score ranged from − 36 to + 12, and participants were categorized according to the prescribed cut-offs described for the assessment. Questions included in the assessment pertained not only to appetite per se, but also included attitudes towards foods, body perception and symptoms associated with anorexia including pain, satiety, and vomiting. Statistical inferences were applied to investigate the differences between cases and controls and to define and classify the severity of anorexia, specifically within the cases.

### Quality of life assessment

The European Organization for the Research and Treatment of Cancer Quality of Life–C30 Questionnaire (EORTC QLQ-C30) Version 3 questionnaire, validated for use in patients with cancer cachexia [21], was self-administered by all participants to assess QoL. Permission to use this assessment was applied for and granted from the EORTC. Scoring was done according to the guidelines prescribed in the EORTC scoring manual [22]. Five functional scales (physical, role, cognitive, emotional, and social) (QL-FS), three symptom scales (fatigue, pain, and nausea and vomiting) (QL-SS), and global health status (QL-G) identified the categories used for statistical investigations.

### Cachexia assessment

Cachexia status was assessed using a five-factor cachexia scoring system [10] inclusive of percentage weight loss in the past 6 months, strength, assistance with walking, rise from a chair, climb stairs and falls (SARC-F), Eastern Cooperative Oncology Group performance status assessment (ECOG), appetite loss, and abnormal biochemistry. Scores were used to categorize cases and controls into no cachexia, pre-cachexia, cachexia, and refractory cachexia categories according to the cut-offs prescribed by the scoring tool.

### Statistical analysis

A sample size calculation, using a one-way analysis of variance (ANOVA) calculation was applied to achieve a 90% power (effect size of 0.52). A hypothesis test of equal means defined a significance level of 5%.

Data Science Workbench, Version 14. Microsoft Excel was used to capture the data, which was imported to STATISTICA 13, TIBCO Software Inc. (2020) for statistical analyses. Summary statistics and descriptive statistics were used to describe the variables. Means were used as the measure of a central location for ordinal and continuous responses and quartiles and standard deviations as indicators of spread, respectively. Correlations between two continuous variables were measured with the Pearson correlation, or Spearman correlation. The relation between discrete variables was investigated with contingency tables and chi-square tests.

Continuous variables were compared between the two groups using ANOVA or equivalently pooled *t* tests. If the variances of the two groups differed significantly, the Welch-test was used. If the residuals from ANOVA were not normally distributed, the Mann–Whitney test was used as the non-parametric equivalent of the pooled *t* test.

For investigational markers and the measure of inflammation, where are there no formal accepted reference values, cut-offs yielded from receiver operating characteristic (ROC) curve analysis were applied to test for significance.

### Ethical clearance

Ethics approval to conduct the study was obtained from the Stellenbosch University Health Research Ethics Committee (HREC) (Ethics approval S19/10/223). All participants gave written informed consent to participate in the study. The study was performed in accordance with the ethical standards as laid down in the 1964 Declaration of Helsinki and its later amendments or comparable ethical standards.

## Results

### Baseline investigations

#### Participant characteristics

Participant characteristics inclusive of the primary diagnoses of the cases are shown in Table 1. The majority of the population was male (*n* = 52, 65%), and the main primary diagnoses were lung, colon, rectal, and stomach cancers with seventy percent of the participants above the age of 60 years, with a mean age of 64.03 years.

**Table 1:** Participant characteristics

	Total participants (*n* = 80)		
Male	52		65%
Female	28		35%
	**Cases**	**Controls**	***p* value**
Age (years)	64.13 (± 12.88)	63.95 (± 12.54)	*p* = 0.95
Males	64.12 (± 11.61)	64.77 (± 11.30)	
Females	64.12 (± 15.43)	62.43 (± 14.92)	
Weight (kg)	68.46 (± 16.13)	81.18(± 13.12)	*p* < 0.01
Total reported weight loss (%)	16.15 (± 8.40)		
Males	14.50 (± 6.70)		
Females	19.21 (± 10.40)		
Body mass index (BMI) (kg/m^2^)	24.00 (± 4.50)	28.46 (± 3.74)	*p* < 0.01
Primary diagnosis	**Males (%)**	**Females (%)**	**Cases (%)**
Lung	5 (19.2%)	3 (21.4%)	8 (20%)
Colon	4 (15.4%)	2 (14.3%)	6 (15%)
Rectal	2 (7.7%)	2 (14.3%)	4 (10%)
Stomach	2 (7.7%)	1 (7.1%)	3 (7.5%)
Breast		2 (14.3%)	2 (5%)
Melanoma	1 (3.8%)	1 (7.1%)	2 (5%)
Mesothelioma	2 (7.7%)		2 (5%)
Mixed diagnoses	10 (38.5%)	3 (21.5%)	13 (32.5%)

Mixed cancer diagnoses include ear (*n* = 1), renal (*n* = 2), brain (*n* = 1), pancreatic (*n* = 1), ovarian (*n* = 1), myloma (*n* = 1), cholangioma (*n* = 1), testicular (*n* = 1), bladder (*n* = 1), lymphoma (*n* = 1), esophageal (*n* = 1), tongue (*n* = 1)

#### Biomarker investigation

As described previously [19], measurement of systemic biomarker concentrations revealed statistically significant increases (*p* < 0.05) in all of the following biomarkers in the group of cancer patients relative to controls: blood platelets, NLR, PLR, SII, CRP, IL-6, IL-8, and TNFα. Concentrations of biomarkers that were significantly decreased (*p* < 0.05) in the cohort of cancer patients included albumin, Hb, and lymphocyte count, while white blood cell counts (WBCs), neutrophil counts, and concentrations of CXCL5 and H3Cit were comparable between patients and controls.

#### Appetite assessment

Raw scores from the FAACT A/CS-12 questionnaire showed that the cases scored significantly lower FAACT A/CS-12 scores than the controls (*p* < 0.01), which remained consistent (*p* < 0.01) between males and females for the groups.

Using prescribed categories for raw appetite scores [poor (− 36 to − 20), moderate (− 19 to − 4) or good (− 3 to + 12)], 48% of cases scored in the “moderate” category, 30% of the cases scored “poor” appetites, and 22% reported “good” appetite scores. In contrast to this, 95% of controls scored “good” appetites, with only 5% indicating moderate appetites (Supplement Fig. 1). No controls scored poor appetites.

#### Quality of life investigations

For QoL assessment, QL-G, QL-FS, and QL-SS all showed significant differences in scores between cases and controls (*p* < 0.01). The cases scored lower values than the controls for QL-G (41.04 versus 83.75) and QL-FS (57.33 versus 91.58) respectively, indicating a poorer QoL and for QL-SS, the cases scored higher (40.29) than the controls (7.68), indicating the presence of greater symptoms. (Supplement Table 1).

Using prescribed EORTC QLQ-C30 reference values for cases (specific reference values chosen from the EORTC QLQ-C30 reference manual [23] pertaining to cancer patients presenting with stage 3 or stage 4 cancer) results showed that the cancer patients demonstrated significantly “worse” QoL scores compared to the prescribed reference values for the categories QL-G (*p* < 0.01), QL-FS (*p* < 0.01), and QL-SS (*p* = 0.01).

#### Cachexia staging

For all cases and controls, the mean cachexia scores were significantly different (*p* < 0.01). No controls scored sufficient points to be classified as cachectic; however, the results showed that 60% (*n* = 24) of cases were classified as cachectic, 22.5% (*n* = 9) with pre-cachexia, and 17.5% (*n* = 7) of cases presented with refractory cachexia (Supplement Fig. 2).

### Statistical relationships of biomarkers to quality of life, appetite, and cachexia

#### Appetite

**Fig. 1:**
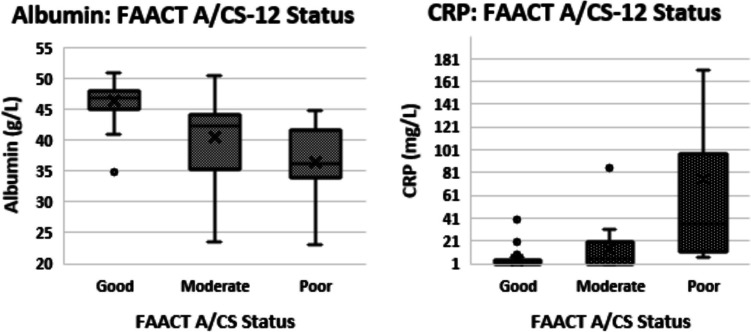
Relationships of albumin and C-reactive protein (CRP) to functional assessment of anorexia/cachexia therapy (FAACT A/CS-12) categories. Abbreviations: C-reactive protein (CRP), functional assessment of anorexia/cachexia therapy (FAACT A/CS-12)

Significant relationships differed according to categories applied to the variables and methods of statistical analysis applied. FAACT A/CS-12 scores showed significant associations with NLR (*p* = 0.002), Hb (*p* < 0.001), and PLR (*p* < 0.01), using continuous variables for both FAACT A/CS-12 scores and biomarkers. Using categories for appetite scores as “good”, “moderate”, and “poor”, albumin (*p* = 0.03) and CRP (*p* = 0.002) were significantly associated with appetite (Fig. 1).

#### Quality of life

In the investigation of relationships of QoL to biomarkers of cachexia, the results (Table 2) showed that albumin, NLR, Hb, PLR, SII, TNFα, IL-8, and CRP were all significantly related to QL-G, QL-FS, and QL-SS aspects of QoL assessment. In contrast, WBC, neutrophils, CXCL5, IL-6, and H3Cit showed no significant relationships to any QoL sectors.

**Table 2:** Summary of relationships of quality of life with blood markers

Marker	QL-G		QL-FS		QL-SS	
	Correlation coefficient	*p* value	Correlation coefficient	*p* value	Correlation coefficient	*p* value
Albumin	0.633	** < 0.001**	0.630	** < 0.001**	− 0.604	** < 0.001**
WBC	− 0.067	0.538	0.001	0.992	0.051	0.651
Neutrophils	− 0.109	0.335	− 0.028	0.803	0.093	0.414
Lymphocytes	0.279	**0.012**	0.212	0.059	− 0.261	**0.020**
NLR	− 0.381	** < 0.001**	− 0.267	**0.017**	0.346	**0.002**
Hb	0.665	** < 0.001**	0.656	** < 0.001**	− 0.642	** < 0.001**
Platelets	− 0.312	**0.005**	− 0.177	0.116	0.227	**0.043**
PLR	− 0.533	** < 0.001**	− 0.491	** < 0.001**	0.551	** < 0.001**
SII	− 0.378	** < 0.001**	− 0.287	**0.010**	0.360	**0.001**
CXCL5	− 0.173	0.125	− 0.027	0.811	0.130	0.250
TNFα	− 0.510	** < 0.001**	− 0.456	** < 0.001**	0.522	** < 0.001**
IL-6	− 0.180	0.112	− 0.217	0.053	0.161	0.153
IL-8	− 0.251	**0.025**	− 0.298	**0.007**	0.274	**0.014**
CRP	− 0.504	** < 0.001**	− 0.457	** < 0.001**	0.494	** < 0.001**
H3Cit	0.105	0.354	0.109	0.338	− 0.106	0.345

*H3Cit* citrullinated histone H3; *CRP* C-reactive protein; *CXCL5* C-X-C motif chemokine ligand 5; *Hb* hemoglobin; *IL-6* interleukin-6; *IL-8* interleukin-8; *NLR* neutrophil to lymphocyte ratio; *PLR* platelet to lymphocyte ratio; *QL-G* quality of life global; *QL-FS* quality of life functional scales; *QL-SS* quality of life symptom scale; *SII* systemic immune inflammation index; *TNFα* tumor necrosis factor alpha; *WBC* white blood cell count

**Fig. 2:**
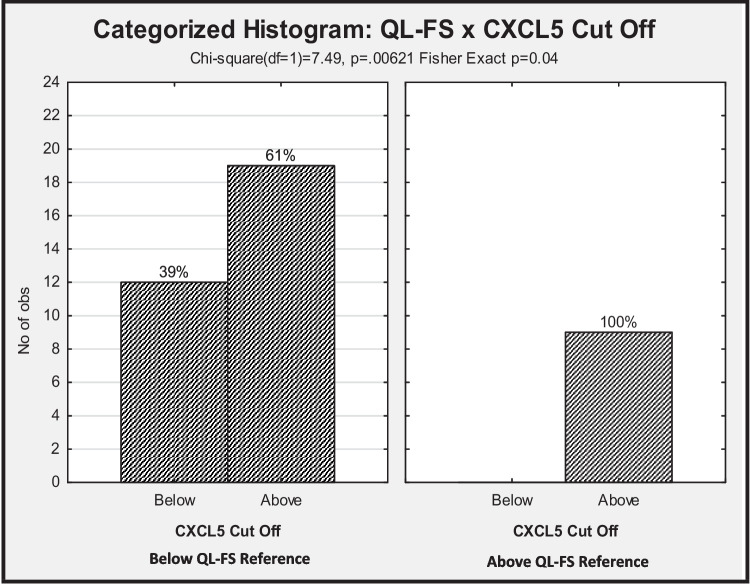
Significant relationship of C-X-C motif chemokine ligand 5 (CXCL5) to quality-of-life functional Scales (QL-FS). Abbreviations: C-X-C motif chemokine ligand 5 (CXCL5); Quality of life functional scales (QL-FS)

Negative significant correlations were found for QL-G and QL-FS with inflammatory biomarkers, indicating higher QL-G and QL-FS scores, in the presence of lower markers of inflammation and the opposite relationship (statistically significant positive correlations) was found for albumin and Hb. No relationships were found between QL-G and QL-FS and the investigational markers CXCL5 and H3Cit; however, when cut-offs for biomarkers and QL-FS were applied, CXCL5 showed a significant association with QL-FS (*p* = 0.04) (Fig. 2).

#### Cachexia

Several of the biomarkers were found to be significantly correlated with the cachexia stage scoring (CSS) scores as can be seen in Table 3. Those that showed positive correlations included NLR, PLR, SII, TNFα, IL-6, IL-8, and CRP. Significant negative correlations were noted for albumin, Hb, and lymphocyte count. No significant correlations were found for neutrophils, platelets, WBC, CXCL5, or H3Cit.

**Table 3:** Cachexia stage scoring scores correlations to biomarkers

Biomarker	Significance	Correlation coefficient
Albumin	*p* < 0.01	*r* = − 0.69
Lymphocytes	*p* = 0.02	*r* = − 0.27
NLR	*p* < 0.01	*r* = 0.41
Hb	*p* < 0.01	*r* = − 0.71
PLR	*p* < 0.01	*r* = 0.53
SII	*p* < 0.01	*r* = 0.4
TNFα	*p* < 0.01	*r* = 0.56
IL-6	*p* = 0.03	*r* = 0.24
IL-8	*p* < 0.01	*r* = 0.3
CRP	*p* < 0.01	*r* = 0.59

*CRP* C-reactive protein; *Hb* hemoglobin; *IL-6* interleukin-6; *IL-8* interleukin-8; *NLR* neutrophil to lymphocyte ratio; *PLR* platelet to lymphocyte ratio; *SII* systemic immune inflammation index; *TNFα* tumor necrosis factor alpha

## Discussion

When comparing baseline assessments of anorexia, QoL, cachexia, and biomarkers of patients with advanced cancer to healthy controls significant differences for most parameters were evident. These differences provided the foundation for further investigations of the relationships of anorexia, QoL, and cachexia to both previously researched and emerging biomarkers with the primary objective of advancing knowledge of potential means to mitigate the catastrophic sequelae of cancer cachexia.

In the current study, the FAACT A/CS-12 tool clearly demonstrated the stark difference in anorexia scores (cases versus controls (*p* < 0.01)) that included questions pertaining to attitude towards foods, body weight perception and appetite in general. Only 22% of cases were classified as having “good” appetites, while 30% of cases were classified as having “poor” appetites. These results are similar to those in other studies which reported prevalences of 39% [24] and 41% [25] with “poor” appetites respectively.

For all three domains of QoL assessments, QL-G, QL-FS, and QL-SS, the EORTC QLQ-C30 scores for the cases were significantly different from the prescribed reference values of the EORTC QLQ-C30 QoL scoring manual. The link between QoL and cachexia is supported in the literature and with the worldwide rise of mental health disorders and depression [12], being equally important in cachexia evaluations. In the current study, investigations took place during the height of the COVID-19 pandemic, when nationwide “lockdowns” exacerbated loneliness, isolation, anxiety, and depression with vast negative effects on mental health overall, which may have yielded unexpected lower QoL scores.

The comprehensive cachexia assessment utilized in the current study confirmed the presence of cachexia in the cases. Sixty percent of the cases were categorized as having cachexia, 17.5% presented with refractory cachexia and 9.5% with pre-cachexia. These results align with other research where between 15 and 40% of patients with cancer present with cachexia [26]. Considering that cachexia alone may be the cause of death in more than 20% of patients and that cachexia may occur in approximately 80% of patients with advanced illness [26], assessments of cachexia are warranted in clinical cancer research to abate the long-term decline with progressing cachexia.

There are many different components that contribute to the symptoms of anorexia in cancer patients including pro-inflammatory cytokines, tumors directly resulting in dysphagia or altering gut function, zinc deficiency, the side-effects of cancer treatment, depression, and pain [27]. Anorexia identification, as part of baseline cachexia assessments, has been shown to be an independent indicator of survival, even more so than weight loss [28]. Furthermore appetite loss is linked to a reduced QoL, reduced tolerability to cancer treatment and ultimately a reduced prognosis [25].

Other factors that may affect QoL scores include the nature of primary diagnoses and time of diagnosis in the trajectory of the disease process. Research has found that diagnosis and treatment of rectal cancers where colostomy bags are necessary, may cause increased psychological distress [29]. Ten percent of the cases in the current study presented with rectal cancer which may explain, in part, the poor QoL score results. Diagnoses made when the patient’s disease is advanced will result in relatively poorer QoL scores. In the current study, 20% of the cases presented with lung cancer, the diagnosis of which, in contrast to other primary diagnoses, usually occurs when the disease is more advanced [29], therefore potentially further explaining the relatively poor QoL results.

Daly et al. used the EORTC QLQ-C30 and graded patients according to BMI and weight loss using the 5 × 5 matrix analysis by Martin et al. [30]. Their patients had similar ages and primary diagnoses (gastrointestinal cancers and lung cancer) to the patients in the current study. They showed that the matrix grading system could identify patients at risk of poorer QoL, poor prognosis, and increased symptom burden [31]. The mean reported percentage weight loss of 16% in the cases recruited to this study together with the category grading of 4, according to the weight loss grading system, supports the prevalence of poor QoL scores demonstrated in the current study.

Baseline blood analyses showed that routine hematological and blood chemistry markers, cytokines, and biomarkers of inflammation were significantly different between cases and controls [19]. Hypoalbuminemia has been reported to have a direct impact on caregiver burden and is closely linked to a compromised QoL [32]. The fatigue, related to anemia (reduced Hb), also has a negative effect on QoL and overall wellbeing [33]. The significantly low Hb and albumin present in the patients may therefore be indicative of contributing factors to the compromised QoL scores observed in this study.

Significant differences were found between cases and controls for lymphocytes, platelets, NLR, PLR, and SII, and further significance was evident when comparing the mean values to the ROC curve analysis reference values. Additionally, CRP, TNFα, IL-6, and IL-8 were significantly elevated in the cases [19]. Importantly, research into the association of IL-8 with distant metastases, tumor progression, and tumor stage renders IL-8 useful as a diagnostic tool to determine the progression status of cancer. This is particularly relevant where QoL is concerned, as raised IL-8 levels may indicate extensive metastatic disease, and therefore direct the treatment plan to be either aggressive or palliative [34].

In contrast to other studies, that showed more than a threefold increase of serum CXCL5 concentrations in cancer [17], the current study found no difference for cases vs. controls for CXCL5 (*p* = 0.22) [19]. Studies that report raised CXCL5 levels may be questionable due to inconsistencies in CXCL5 measurement, differing primary diagnoses [17] and the degree of metastatic spread [35], factors that may have influenced CXCL5 results in the study by Lipshitz et al. [19]. Additionally, no significance for H3Cit was found between the cases and controls (*p* = 0.99) [19], potentially indicating the absence of systemic neutrophil activation and neutrophil extracellular trap (NET) formation being a source of circulating H3Cit in cancer patients [18] with only one of 40 case participants demonstrating a raised neutrophil content.

The significant differences of anthropometry, appetite, QoL, cachexia status, cytokines, and inflammatory biomarkers between the cases and controls, further supported the underlying pathophysiology of cancer cachexia at play, a phenomenon that is supported in the literature [36]. These differences supported the advanced nature of the disease process and provided a strong baseline from which to pursue meaningful correlations to QoL, appetite, and cachexia to the biomarkers.

The routine biomarkers appeared to be the most reliable in showing significant relationships to QoL and appetite. Regarding QoL, albumin significantly predicted poorer scores in all three facets of the EORTC QLQ-C30 with positive correlations to both QL-G, and QL-FS and negative correlations to QL-SS. These results are supported in other research, albeit that different tools were used to assess QoL [37]. In the current study, a lower serum albumin was also found to be indicative of poorer FAACT A/CS-12 scores similar to findings in the literature [38]. Reduced albumin is synonymous with increased inflammation and may therefore be useful in predicting the extent of cachexia in cancer patients [39].

Functional and cognitive decline, depression, and poor QoL are all associated with low Hb levels [40]. Cancer-related fatigue (CRF) is well described in the literature in cancer patients and may be related to anemia, together with loss of appetite and lethargy [41]. This relationship was confirmed in the current study where Hb was significantly related to QoL and appetite scores. Hemoglobin measurement is deemed extremely useful as a marker to predict malnutrition, while conversely malnutrition can be used to indicate the presence of anemia [42].

There are very few studies, if any, that have made direct associations between NLR, PLR, and SII with FAACT A/CS-12 assessment, EORTC QLQ-C30 assessment or cachexia status. The combination of tools used in this study is therefore unique. Regarding cachexia status, most studies define cachexia using percentage weight loss (2% or 5% weight loss), and have demonstrated that cachexia correlated to WBC, CRP, and NLR [43]. The comprehensive cachexia scoring tool used in this study revealed that NLR, PLR, and SII were positively and significantly associated with cachexia status, indicating that research outcomes may be dependent on the tools utilized to define the parameters being examined. For QoL and appetite, PLR, NLR, and SII showed significant associations to these assessments. More specifically, for QoL, albumin, lymphocytes, and Hb showed negative correlations to QL-SS (increased symptoms for lower biomarkers), such trends that were confirmed by Cordeiro et al. [44], who recommended PLR, as a primary screening test to identify a compromised nutritional status, QoL and reduced appetite.

Studies using experimental models show clearly that the proinflammatory cytokines, including IL-1, IL-6, and TNFα are intermediaries of anorexia [45] and that fatigue, poor functionality, and a poor QoL are also factors related to these inflammatory markers, predicting overall survival and prognosis [44]. Additionally, CRP, with its reliability for prognosis forecasting in cancer [44], has been confirmed to be significantly and positively associated with several fields in the EORTC QLQ-C30 assessment (fatigue, nausea, and vomiting, pain, dyspnea, insomnia, appetite loss, diarrhea, and financial difficulty), while being significantly and negatively associated with physical functioning, role functioning, cognitive functioning, social functioning, and global QoL [46]. The relationship of CRP to appetite scores is confirmed both in the current study (*p* = 0.002) and by other research that established raised CRP significantly related to deteriorating appetite.

According to the literature, both IL-6 and IL-8, using various measurement tools, showed significant associations with QoL categories including pain, depression, and fatigue [47]. Similarly in the current study, CRP, TNFα, IL-6, and IL-8 were all significantly related to QL-G, QL-FS, and QL-SS, supporting this relationship and the utility of cachexia biomarkers in predicting QoL and conversely the potential of QoL assessments in predicting the expression of pro-inflammatory cytokines, systemic inflammation, and extent of disease. A reduced appetite has been shown to be linked to increased expression of IL-1, TNFα, and IL-6, with an increase in IL-6 being the most prominent biomarker to indicate of appetite loss [47]. These findings have the potential to enable goal-directed and individualized treatment.

There appears to be a paucity of literature regarding the relationships of both CXCL5 and H3Cit to QoL and appetite in cancer cachexia. Although no significant relationships for H3Cit to QoL, appetite or cachexia were found in the outcomes of this study, using CXCL5 cut-offs according to ROC curve analysis and QL-FS categories, CXCL5 was shown to be statistically significant with respect to this domain of QoL assessment (*p* = 0.04). No correlations were found for these markers to QoL or appetite where continuous variables were used; however, further research of CXCL5 to QoL may be warranted. The results of this study form a foundation for future research to better define the integration of CXCL5 and H3Cit as biomarkers in cancer cachexia management and to provide a paradigm of thought on how to combine the tools of cachexia, appetite, and QoL measurement to be standardized for future research.

The current study had several limitations. These included multiple primary diagnoses of cases and the relatively small sample size that is under-powered for subset statistical analyses, as well as the fact that the study was conducted in a single private oncology setting, which may have confounded outcomes of the QoL assessment as the patient cohort represented a specific socioeconomic group. There is also data to suggest that while cachexia is typically associated with the more advanced stages of cancer, cancer stage, and cachexia status may be separable at a molecular level [8], therefore further complicating standardization of investigations. Furthermore, future studies should compare QoL and biomarkers in advanced cancer patients with and without cachexia.

Additional recommendations for future research are to apply the principles and tools used in the current study to other novel biomarkers including GDF-15, FGF-21, fractakline, IFN-gamma, IL-10, IL-16, M-CSF, and MPIF-1 (CCL23) and transforming growth factor β1 to be investigated in both cachectic and non-cachectic cancer patients. The long-term goal of such research is to identify new therapies to improve appetite and satiety, and to reduce skeletal muscle mass (which will ultimately improve QoL) [13]. This approach will improve current understandings of the relationships between the less well understood biomarkers of advanced cancer, QoL, appetite, and cachexia so that treatment interventions can be targeted to decrease the incidence of cachexia in advanced cancer patients.

### Supplementary Information

Below is the link to the electronic supplementary material.

Supplementary file1 (PDF 64 KB)

Supplementary file2 (PDF 70 KB)

Supplementary file3 (PDF 56 KB)

## Data Availability

The data that support the findings of this study and related study tools are available from the corresponding author, Melanie Lipshitz (melanielevydietcian@gmail.com), upon reasonable request.

## References

[1] Ryan AM, Power DG, Daly L, Cushen SJ, Ní Bhuachalla E, Prado CM (2016). Cancer-associated malnutrition, cachexia and sarcopenia: the skeleton in the hospital closet 40 years later. Proc Nutr Soc.

[2] Srdic D, Plestina S, Sverko-Peternac A, Nikolac N, Simundic AM, Samarzija M (2016). Cancer cachexia, sarcopenia and biochemical markers in patients with advanced non-small cell lung cancer—chemotherapy toxicity and prognostic value. Support Care Cancer.

[3] Argilés JM, Busquets S, López-Soriano FJ (2019). Cancer cachexia, a clinical challenge. Curr Opin Oncol.

[4] Abraham M, Kordatou Z, Barriuso J, Lamarca A, Weaver JM, Cipriano C, Papaxoinis G, Backen A, Mansoor W (2019). Early recognition of anorexia through patient-generated assessment predicts survival in patients with oesophagogastric cancer. PLoS ONE.

[5] Ryan AM, Prado CM, Sullivan ES, Power DG, Daly LE (2019). Effects of weight loss and sarcopenia on response to chemotherapy, quality of life, and survival. Nutrition.

[6] Blauwhoff-Buskermolen S, Ruijgrok C, Ostelo RW, de Vet HCW, Verheul HMW, van der Schueren MAE, Langius JAE (2016). The assessment of anorexia in patients with cancer: cut-off values for the FAACT–A/CS and the VAS for appetite. Support Care Cancer.

[7] Ribaudo JM, Cella D, Hahn EA, Lloyd SR, Tcheckmedyian NS, Von Roenn J, Leslier WT (2000). Re-validation and shortening of the functional assessment of anorexia/cachexia therapy (FAACT) questionnaire. Qual Life Res.

[8] Narasimhan A, Shahda S, Kays JK, Perkins SM, Cheng L, Schloss KNH, Schloss D (2020). Identification of potential serum protein biomarkers and pathways for pancreatic cancer cachexia using an aptamer-based discovery platform. Cancers.

[9] Davis M, Yavuzsen T, Kirkova J, Walsk D, Karafa M, LeGrand S, Lagman R (2009). Validation of a simplified anorexia questionnaire. J Pain Symptom Manage.

[10] Zhou T, Wang B, Liu H, Yang K, Thapa S, Zhang H, Li L, Yu S (2018). Development and validation of a clinically applicable score to classify cachexia stages in advanced cancer patients. J Cachexia Sarcopenia Muscle.

[11] Aaronson NK, Ahmedzai S, Bergman B, Bullinger M, Cull A, Duez N, Filiberti A, Fletchner H (1993). The European Organization for Research and Treatment of Cancer QLQ-C30: a quality-of-life instrument for use in international clinical trials in oncology. J Natl Cancer Inst.

[12] McDonald JJ, Fallon MT, Laird BJA (2019). Meaningful measures in cancer cachexia: implications for practice and research. Curr Opin Support Palliat Care.

[13] Kadakia KC, Hamilton-Reeves JM, Baracos VE (2023) Current therapeutic targets in cancer cachexia: a pathophysiologic approach. Am Soc Clin Oncol Educ B 43. 10.1200/edbk_389942 10.1200/EDBK_389942 PMC11019847 37290034

[14] Baracos VE, Martin L, Korc M, Guttridge CD, Fearon KCH (2018). Cancer-associated cachexia. Nat Rev Clin Oncol.

[15] Roca H, Jones JD, Purica MC, Weidner S, Koh AJ, Kuo R, Wilkinson JE, Wang Y (2018). Apoptosis-induced CXCL5 accelerates inflammation and growth of prostate tumor metastases in bone. J Clin Invest.

[16] Zhang W, Wang H, Sun M, Deng X, Wu X, Ma Y, Li M, Shuoa S, You Q, Miao L (2020). CXCL5 / CXCR2 axis in tumor microenvironment as potential diagnostic biomarker and therapeutic target. Cancer Commun.

[17] Wu K, Yu S, Liu Q, Bai X, Zheng X, Wu K (2017). The clinical significance of CXCL5 in non-small cell lung cancer. Onco Targets Ther.

[18] Thålin C, Lundstrom S, Seignez C, Daleskog M, Lundstrom A, Henriksson P, Helleday T, Phillipson M, Wallen H, Demers M (2018). Citrullinated histone H3 as a novel prognostic blood marker in patients with advanced cancer. PLoS ONE.

[19] Lipshitz M, Visser J, Anderson R, Nel DG, Smit T, Steel HC, Rapoport BL (2023). Emerging markers of cancer cachexia and their relationship to sarcopenia. J Cancer Res Clin Oncol.

[20] Citrullinated Histone H3 (Clone 11D3) ELISA Kit. no. 501620 (2020) Cayman chemical company, 1180 E Ellsworth Road, Ann Arbor MI, USA. https://www.caymanchem.com/product/501620/citrullinated-histone-h3-(clone-11d3)-elisa-kit. Accessed 10 Jun 2021

[21] Mystakidou K, Tsilika E, Parpa E, Kalaidopoulou O, Smyrniotis V, Vlahos L (2001). The EORTC core quality of life questionnaire (QLQ-C30, version 3.0) In terminally ill cancer patients under palliative care: validity and reliability in a hellenic sample. Int J Cancer.

[22] Fayers P, Aaronson N, Bjordal K, Groenvold M, Curran D, Bottomley A (2001) EORTC QLQ- C30 scoring manual. European organisation for research and treatment of cancer, 3rd ed. Brussels, Belgium

[23] Scott NW, Fayers PM, Aaronson NK, Bottomly A, de Graeff A, Groenvold M, Grundy C, Koller M, Petersen M, Sprangers MA. MAG, EORTC Quality of Life Group (2008) EORTC QLQ- C30 reference values manual. 2nd edn, EORTC Quality of Life Group. Brussels, Belgium. https://groups.eortc.be/qol/downloads/reference_values_manual2008.pdf. Accessed 26 Jun 2021

[24] Franceschini JP, Jamnik S, Santoro IL (2020). Role that anorexia and weight loss play in patients with stage iv lung cancer. J Bras Pneumol.

[25] Muscaritoli M, Lucia S, Farcomeni A, Lorusso V, Saraconi V, Barone C, Plastoni F (2017). Prevalence of malnutrition in patients at first medical oncology visit: the PreMiO study. Oncotarget.

[26] Mondello P, Lacquaniti A, Mondello S, Bolignano D, Pitni V, Aloisi C, Beumi M (2014). Emerging markers of cachexia predict survival in cancer patients. BMC Cancer.

[27] Ezeoke CC, Morley JE (2015). Pathophysiology of anorexia in the cancer cachexia syndrome. J Cachexia Sarcopenia Muscle.

[28] Sundstrøm S, Bremnes RM, Brunsvig P, Aasebø U, Kaasa S (2006). Palliative thoracic radiotherapy in locally advanced non-small cell lung cancer: can quality-of-life assessments help in selection of patients for short- or long-course radiotherapy?. J Thorac Oncol.

[29] Pasetto LM, Falci C, Compostella A, Sinigaglia G, Rossi E, Monfardini E (2007). Quality of life in elderly cancer patients. Eur J Cancer.

[30] Martin L, Senesse P, Gioulbasanis I, Antoun S, Bozzetti F, Deans C, Strasser F (2015). Diagnostic criteria for the classification of cancer-associated weight loss. J Clin Oncol.

[31] Daly L, Dolan R, Power D, Ni Bhuachalla EN, Sim W, Fallon M, Cushen S (2020). The relationship between the BMI-adjusted weight loss grading system and quality of life in patients with incurable cancer. J Cachexia Sarcopenia Muscle.

[32] Bullock AF, Greenley SL, McKenzie GAG, Paton LW, Johnson MJ (2020). Relationship between markers of malnutrition and clinical outcomes in older adults with cancer: systematic review, narrative synthesis and meta-analysis. Eur J Clin Nutr.

[33] Knight K, Wade S, Balducci L (2004). Prevalence and outcomes of anemia in cancer: a systematic review of the literature. Am J Med.

[34] Paczek S, Łukaszewicz-Zajac M, Gryko M, Mroczko P, Kulczyńska-Przybik A, Mroczko B (2020). CXCL-8 in preoperative colorectal cancer patients: significance for diagnosis and cancer progression. Int J Mol Sci.

[35] Lim JB, Chung HW (2015). Serum ENA78/CXCL5, SDF-1/CXCL12, and their combinations as potential biomarkers for prediction of the presence and distant metastasis of primary gastric cancer. Cytokine.

[36] Mantovani G, Maccio A, Mura L, Massa E, Mudu MC, Mulas C, Lusso MR, Madeddu DA (2000). Serum levels of leptin and proinflammatory cytokines in patients with advanced-stage cancer at different sites. J Mol Med.

[37] Guo ZQ, Yu JM, Li W, Fu ZM, Lin Y, Shi YY, Hu W, Ba Y, Li SY (2019). Survey and analysis of the nutritional status in hospitalized patients with malignant gastric tumors and its influence on the quality of life. Support Care Cancer.

[38] Wang B, Thapa S, Zhou T, Liu H, Li L, Peng G, Yu S (2020). Cancer-related fatigue and biochemical parameters among cancer patients with different stages of sarcopenia. Support Care Cancer.

[39] Fiala O, Pesek M, Finek J, Racek J, Minarik M, Benesova L, Bortlicek Z (2016). Serum albumin is a strong predictor of survival in patients with advanced-stage non-small cell lung cancer treated with erlotinib. Neoplasma.

[40] Tan T, Ong WS, Rajasekaran T, Koo KN, Chan LL, Poon D, Chowdury AR, Krishna L, Kanesvaran R (2016). Identification of comprehensive geriatric assessment based risk factors for malnutrition in elderly Asian cancer patients. PLoS ONE.

[41] Lind M, Vernon C, Cruickshank D, Wilkinson P, Littlewood T, Stuart N, Jenkinson C (2002). The level of haemoglobin in anaemic cancer patients correlates positively with quality of life. Br J Cancer.

[42] Wu M, Lian XJ, Jia JM, Cao WT, Yan N, Xin YM, Liu ZR (2019). The role of the patient-generated subjective global assessment (PG-SGA) and biochemical markers in predicting anemia patients with cancer. Support Care Cancer.

[43] Takeda T, Sasaki T, Suzumori C, Mie T, Furukawa T, Yamada Y, Kasuga A (2021). The impact of cachexia and sarcopenia in elderly pancreatic cancer patients receiving palliative chemotherapy. Int J Clin Oncol.

[44] de Cordeiro LDAF, Silva TH, de Oliveira LC, Neto JFN (2020). Systemic inflammation and nutritional status in patients on palliative cancer care: a systematic review of observational studies. Am J Hosp Palliat Med.

[45] Kim EY, Kim YS, Seo J, Park I, Ahn AK (2016). The relationship between sarcopenia and systemic inflammatory response for cancer cachexia in small cell lung cancer. PLoS ONE.

[46] Li L, Chan SL, Mo F, Hui EP, Koh J, Chan AK, Nls Tang, Chu CM, Hui J (2019). Status of inflammation in relation to health related quality of life in hepatocellular carcinoma patients. Qual Life Res.

[47] Paulsen Ø, Laird B, Aass N, Lea T, Fayers KS, Klepstad P (2017). The relationship between pro-inflammatory cytokines and pain, appetite and fatigue in patients with advanced cancer. PLoS ONE.

