# Supplementary material for: Relationships of emerging biomarkers of cancer cachexia with quality of life, appetite, and cachexia
Source: Support Care Cancer. 2024 May 14;32(6):349. doi: 10.1007/s00520-024-08549-5 (PMC11093781; doi:10.1007/s00520-024-08549-5)
Supplement: Supplementary file 1 — Supplementary file1 (PDF 64 KB) [file 520_2024_8549_MOESM1_ESM.pdf]

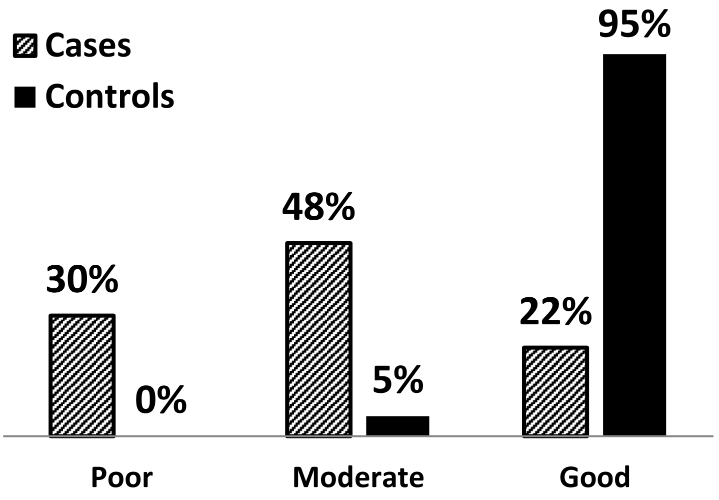

Figure 1 (Supplementary) : Functional assessment of anorexia / cachexia therapy (FAACT A/CS-12) categories
