# Supplementary figures and images for: Relationships of emerging biomarkers of cancer cachexia with quality of life, appetite, and cachexia
Source: Support Care Cancer. 2024 May 14;32(6):349. doi: 10.1007/s00520-024-08549-5 (PMC11093781; doi:10.1007/s00520-024-08549-5)

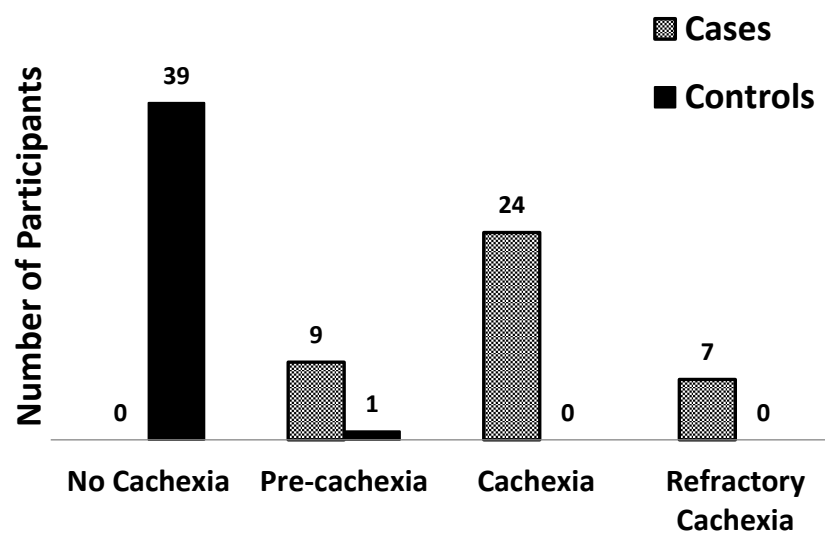

Figure 2: Cachexia categories for cases and controls

Supplement: Supplementary file 2 — Supplementary file2 (PDF 70 KB) [file 520_2024_8549_MOESM2_ESM.pdf]
