# Supplementary material for: Relationships of emerging biomarkers of cancer cachexia with quality of life, appetite, and cachexia
Source: Support Care Cancer. 2024 May 14;32(6):349. doi: 10.1007/s00520-024-08549-5 (PMC11093781; doi:10.1007/s00520-024-08549-5)
Supplement: Supplementary file 3 — Supplementary file3 (PDF 56 KB) [file 520_2024_8549_MOESM3_ESM.pdf]

Table 1 (Supplementary) - European organisation for the research and treatment of cancer quality of life–C30 questionnaire (EORTC QLQ-C30) scores

Category Question	Cases	Controls	Test for Significant difference	Reference Value	p-value for Significance
**Global Health Status (QL-G)**	**41.04 (±16.81)**	**83.75 (±19.42)**	**p < 0.01**	61.5	**p < 0.001**
**Functional scales (QL-FS)**	**57.33 (±20.78)**	**91.58 (±8.58)**	**p < 0.01**	74.32	**p < 0.001**
**Symptom Scales (QL-SS)**	**40.29 (±16.34)**	**7.68 (±7.40)**	**p < 0.01**	18.07	**p = 0.01**
